# Elevated serum cortisol associated with early‐detected increase of brain amyloid deposition in Alzheimer's disease imaging biomarkers among menopausal women: The Framingham Heart Study

**DOI:** 10.1002/alz.70179

**Published:** 2025-04-24

**Authors:** Arash Salardini, Jayandra J. Himali, Muhammad S. Abdullah, Rima Chaudhari, Vanessa Young, Eduardo M. Zilli, Emer R. McGrath, Mitzi M. Gonzales, Emma G. Thibault, Joel Salinas, Hugo J. Aparicio, Dibya Himali, Saptaparni Ghosh, Rachel F. Buckley, Claudia L. Satizabal, Keith A. Johnson, Charles DeCarli, Georges El Fakhri, Ramachandran S. Vasan, Alexa S. Beiser, Sudha Seshadri

**Affiliations:** ^1^ Glenn Biggs Institute for Alzheimer's & Neurodegenerative Diseases UT Health San Antonio San Antonio Texas USA; ^2^ Department of Neurology UT Health San Antonio San Antonio Texas USA; ^3^ NHLBI's Framingham Heart Study Framingham Massachusetts USA; ^4^ Department of Population Health Sciences UT Health San Antonio San Antonio Texas USA; ^5^ Department of Neurology Boston University Chobanian & Avedisian School of Medicine Boston Massachusetts USA; ^6^ Department of Biostatistics Boston University School of Public Health Boston Massachusetts USA; ^7^ Graduate School of Biomedical Sciences UT Health San Antonio San Antonio Texas USA; ^8^ Gonzaba Medical Group (private practice) San Antonio Texas USA; ^9^ HRB Clinical Research Facility University of Galway Galway Ireland; ^10^ School of Medicine University of Galway Galway Ireland; ^11^ Jona Goldrich Center for Alzheimer's and Memory Disorders Neurology Department Cedars‐Sinai Medical Center Los Angeles California USA; ^12^ Department of Radiology Massachusetts General Hospital/Harvard Medical School Boston Massachusetts USA; ^13^ Department of Neurology New York University Grossman School of Medicine New York New York USA; ^14^ Department of Neurology Massachusetts General Hospital/Harvard Medical School Boston Massachusetts USA; ^15^ Center for Alzheimer Research and Treatment Brigham and Women's Hospital Boston Massachusetts USA; ^16^ Department of Neurology University of California at Davis Sacramento California USA; ^17^ Department of Radiology & Biomedical Imaging Yale University New Haven Connecticut USA; ^18^ University of Texas School of Public Health, San Antonio Campus San Antonio Texas USA

**Keywords:** Alzheimer's disease, amyloid deposition, cognitive decline, cortisol, Framingham Heart Study, menopause, PET imaging, sex differences, stress hormones, tau pathology

## Abstract

**INTRODUCTION:**

This study investigates whether midlife cortisol levels predict Alzheimer's disease (AD) biomarker burden 15 years later, with particular attention to sex differences and menopausal status.

**METHODS:**

We analyzed data from 305 cognitively unimpaired Framingham Heart Study participants (48.5% female; mean age: 39.6 ± 8.1 years). Serum cortisol was categorized into tertiles, with amyloid ([^11^C]PiB) and tau ([^18^F]Flortaucipir) positron emission tomography (PET) imaging conducted 15 years later. We performed multivariable regression analyses adjusted for confounders including, apolipoprotein E4 (APOE4) status.

**RESULTS:**

Elevated midlife cortisol correlated with increased amyloid deposition, specifically in post‐menopausal women, predominantly in posterior cingulate, precuneus, and frontal‐lateral regions (*p* < 0.05). No significant associations were observed with tau burden or in males.

**DISCUSSION:**

These findings reveal post‐menopausal women with high midlife cortisol are at increased risk of AD. Results highlight the importance of identifying early risk factors when biomarkers are detectable but cognitive impairment is absent.

**Highlights:**

- High midlife cortisol is linked to increased amyloid deposition in post‐menopausal women.
- Cortisol showed no association with tau pathology.
- Post‐menopausal hormone changes may amplify cortisol's effects on amyloid.

## 1 BACKGROUND

Sporadic Alzheimer's disease (AD) is the leading cause of cognitive decline in older adults. The disease features a prolonged asymptomatic phase of amyloid beta (Aβ) accumulation, eventually triggering tau hyperphosphorylation, widespread neurodegeneration, and progressive cognitive decline.^1^ Recognizing that these biological changes are already well established by the time symptoms emerge, effective early interventions must target AD risk factors during the preclinical stages. However, despite significant advancements in understanding AD pathophysiology, more than half of the overall risk remains unexplained, underscoring the critical need to identify additional AD risk factors that can be targeted during the preclinical stage.^2^

One promising line of investigation centers on cortisol, a steroid hormone essential for cellular homeostasis and the stress response. Genetic studies have identified mutations in glucocorticoid signaling pathways that increase susceptibility to AD.^3^ Several cross‐sectional and longitudinal studies have reported that higher blood cortisol levels are linked to an increased likelihood of developing AD.^4^, ^5^, ^6^, ^7^, ^8^, ^9^, ^10^, ^11^, ^12^, ^13^ Notably, two large studies, the Transdanube Aging Study and the Baltimore Longitudinal Study of Aging, found that elevated serum cortisol predicted an AD diagnosis 7.5 and 11 years later, respectively.^4^, ^5^, ^6^, ^7^ In contrast, another similar study, the Rotterdam Study, did not find a significant association between cortisol and AD risk.^14^

Beyond increasing AD risk, elevated cortisol may accelerate cognitive decline in individuals already positive for amyloid pathology, regardless of their baseline cognitive status.^15^, ^16^, ^17^, ^18^ This acceleration may be due to an exacerbation of existing AD pathology and/or contributions to comorbid pathologies such as vascular comorbidities. Experiments in animal models support this hypothesis: glucocorticoid administration in non‐human primates has been shown to elevate amyloid production,^19^ while blocking glucocorticoid receptors in mouse models with mifepristone yield cognitive and biomarker benefits.^20^

Recent studies offer additional insight into cortisol's role in AD. Mosconi and colleagues investigated the relationship between serum cortisol and AD biomarkers in 277 midlife individuals at risk.^21^ They found that higher cortisol levels were associated with reduced total brain volume, lower glucose metabolism in the frontal cortex, and increased Aβ burden, with more pronounced effects in women, especially post‐menopause. These associations remained significant after adjusting for age, apolipoprotein E4 (APOE4) status, and midlife health factors. In contrast, Wang and colleagues reported no significant correlation between cerebrospinal fluid (CSF) cortisol levels and Aβ concentrations. Instead, they observed positive correlations between CSF cortisol and tau pathology, as well as synaptic degeneration markers like SNAP‐25, suggesting that cortisol may contribute to tau‐related neurodegeneration rather than amyloid accumulation.^16^

To address these gaps and inconsistencies, we conducted a longitudinal analysis using data from the Framingham Heart Study's third‐generation cohort. We assessed the relationship between serum cortisol levels in cognitively unimpaired, middle‐aged individuals and amyloid/tau burdens approximately 15 years later. This approach allowed us to investigate cortisol's impact at an earlier stage of AD pathogenesis, where interventions might be most effective. Given the neuroprotective effects of estrogen and testosterone, which mitigate cortisol's deleterious impact on neural tissues,^22^, ^23^, ^24^ we also explored sex‐specific differences, focusing particularly on post‐menopausal risk. We hypothesized that cortisol's impact on Alzheimer's pathology would be more pronounced in women, especially after menopause, consistent with some previous findings.

## 2 METHODS

### 2.1 Study design

The study protocol received approval from the institutional review boards of Boston University Medical Center and Massachusetts General Hospital. All participants provided informed consent and were enrolled in the Generation 3 (Gen 3) or the corresponding multiethnic OMNI cohort of the Framingham Heart Study (FHS). FHS was established in 1948. It spans three generations of subjects derived from the residents of Framingham MA (Original, Offspring [Gen 2], and Gen 3). One of the widely acknowledged shortcomings of this study is the racial homogeneity of the sample (∼98% were non‐Hispanic White individuals). To make the study more representative of the general United States population, two additional multiethnic cohorts (OMNI 1 and 2) were recruited in 1994 and 2003, respectively.

Gen 3 cohort has undergone three comprehensive assessments: the first quadrennial examination (2002–2005), the second (2008–2011), and the third (2016–2019). Blood samples for serum cortisol measurement were collected during the first exam. Each visit included detailed demographic, socioeconomic, and clinical evaluations through history, physical examination, standardized tools, and laboratory tests. Participants aged 32 to 75 who completed both the first and third exams, had prior brain MRI scans, and were free from neurological conditions (e.g., symptomatic stroke, dementia, brain tumors, multiple sclerosis, or major traumatic brain injury) were eligible for a PET imaging sub‐study. This analysis included 305 individuals with serum cortisol levels from the first exam and follow‐up PET data.

**RESEARCH IN CONTEXT**

1. **Systematic review**: Previous research identified associations between elevated cortisol levels and increased Alzheimer's disease (AD) risk, amyloid‐beta (Aβ) burden, and cognitive decline. However, findings regarding sex‐specific effects and cortisol's relationship with tau pathology have been inconsistent. Large cohort studies like the Baltimore Longitudinal Study of Aging and Transdanube Aging Study found elevated cortisol predictive of AD years later, but other studies, such as the Rotterdam Study, reported no significant associations.
2. **Interpretation**: Using longitudinal data from a very well‐characterized cohort, the Framingham Heart Study, this study uniquely assessed midlife cortisol levels in relation to Aβ and tau burdens 15 years later. Findings highlight sex‐specific effects, showing increased amyloid deposition in post‐menopausal women but no associations in men or with tau pathology. This provides new insights into cortisol's role during early AD pathogenesis. The cognitively unimpaired cohort and reliable positron emission tomography (PET) biomarkers underscore cortisol's role in preclinical AD, despite effects that are modest yet comparable with other well‐studied AD risk in midlife.
3. **Future directions**: This study emphasizes the need for further research into sex‐specific pathways linking cortisol to AD, particularly in post‐menopausal women. Future work should investigate potential interventions targeting cortisol dysregulation during the preclinical stage of AD, using longitudinal cortisol measures and assessing temporal lobe progression.

### 2.2 Assessment of cortisol

Fasting cortisol concentrations were measured in venous blood, typically taken between 7:30 and 9:00 AM after resting the subjects for at least 10 min. The serum was stored at −80°C and later thawed for analysis. Cortisol concentrations were determined using a chemiluminescent immunoassay (Roche Diagnostics, Indianapolis, IN), with intra‐assay coefficients of variation ranging from 3.3% to 10% for high and low concentrations and a detection range of 0.036 to 63.4 µg/dL.^25^

### 2.3 Imaging acquisition

[^11^C]Pittsburgh Compound B (PiB) Aβ and [^18^F]Flortaucipir (FTP) tau PET images were acquired at Massachusetts General Hospital using two scanners: the Siemens ECAT HR+ (*n* = 244) and the five‐ring GE Discovery MI (*n* = 61). PiB imaging followed established protocols, with data collected 40 to 60 min post‐bolus injection of 10 to 15 mCi of radioligand.^26^ FTP images were acquired over 40 to 100 min post‐injection of 9.0 to 11.0 mCi of FTP in 4 × 5‐min frames. Both PiB and FTP images were co‐registered to high‐resolution T1‐weighted brain MRI images using SPM12. To harmonize data from the two scanners, GE Discovery images were smoothed using a 6 mm Gaussian filter.

### 2.4 Amyloid imaging analysis

Key regions of interest (ROIs) for Aβ deposition included the precuneus, posterior cingulate cortex, and cerebellum, identified using FreeSurfer 6.0, as these regions are among the earliest affected by amyloid pathology. An aggregate measure of amyloid deposition, termed the FLR (frontal, lateral, and retro‐splenial cortices), was used for primary analyses. Distribution volume ratios (DVRs) of PiB were calculated with the cerebellar gray matter as the reference region.^27^ The FLR was determined by averaging DVR values across the superior frontal, inferior frontal, rostral middle frontal, rostral anterior cingulate, medial orbitofrontal, inferior and middle temporal, inferior parietal, and precuneus regions.^13^ Amyloid load in the superior temporal sulcus (STS) was included as a secondary endpoint due to its potential as a predictor of future cognitive outcomes.^28^ ROI uptake values were averaged across hemispheres, and volume correction was not applied due to the relatively young age of participants. PiB is a well‐established amyloid PET tracer, and its reliability, while under ongoing evaluation, was supported here by data harmonization across scanners and standardized ROI analysis.^29^

### 2.5 Tau imaging analysis

Primary ROIs for tau imaging included the inferior temporal gyrus, entorhinal cortex, posterior cingulate, and cerebellar gray matter, defined using FreeSurfer 6.0. The standardized uptake volume ratio (SUVR) was calculated using the cerebellar gray matter as the reference.^28^ These primary ROIs correspond to areas initially affected by tau pathology in AD. Secondary SUVR analyses were performed in additional regions prone to tau accumulation, including the fusiform, precuneus, hippocampus, parahippocampal cortex, amygdala, insula, temporal pole, superior frontal orbital gyrus, and rhinal cortex.^30^ FTP's reliability for tau imaging is widely recognized, with our study minimizing variability through MRI co‐registration and scanner harmonization, addressing concerns raised by Therriault et al. (2024).^29^

### 2.6 Statistical analysis

All analyses were conducted using SAS version 9.4 (SAS Institute Inc., Cary, NC). Descriptive statistics were generated to summarize parameters related to the study cohort. To investigate the associations between serum cortisol levels and amyloid/tau PET burden, serum cortisol was used both as a continuous measure (square‐root transformed to normalize the distribution) and was categorized into tertiles to account for potential non‐linear, U‐shaped relationships. Multivariable linear regression was used to assess these associations. For amyloid analyses, Model 1 (M1) adjusted for sex, age at PET imaging, age‐squared, interval between cortisol assessment and PET, and scanner type; Model 2 (M2) added APOE4 status. For tau analyses, Models 1 and 2 followed the same adjustments, with Model 3 (M3) including FLR amyloid burden. Interaction effects (e.g., sex, menopause status) were tested using regression models with interaction terms. No post hoc tests were conducted, as our analyses focused on predefined ROIs and hypotheses. Statistical significance was defined as *p* < 0.05 for primary tests, while interaction terms were considered significant at *p* < 0.10, in line with exploratory analysis conventions from prior FHS publications. Findings are reported overall and by serum cortisol tertiles (Table 1).

**TABLE 1:** Descriptive statistics.

Parameter	All *N* = 305	Tertile 1 (*n* = 101)	Tertile 2 (*n* = 102)	Tertile 3 (*n* = 102)
Cortisol level, µg/dL		< 11.08 µg	11.08–15.50	>15.50
Cortisol, ug/dL, median [quartile1, quartile3]	13.2 [10.1, 17.9]	9.1 [7.5, 10.1]	13.2 [12.0, 14.2]	20.2 [17.9, 24.03]
Female, n (%)	148 (48.5)	56 (18.4)	38 (12.5)	54 (17.7)
Menopause, n (%)	86 (58.1)	37 (66.1)	26 (68.4)	23 (42.6)
Age at Clinic Exam‐1, years (y)	39.6 ± 8.1	41.2 ± 6.7	41.1 ± 8.4	36.7 ± 8.4
Age at PiB PET, years	54.7 ± 8.1	56.4 ± 6.8	56.1 ± 8.5	51.6 ± 8.2
Time of cortisol to PiB PET, y	15.04 ± 1.62	15.1 ± 1.7	15.0 ± 1.7	15.0 ± 1.5
Time cortisol to tau PET, years	14.8 ± 1.6	14.9 ± 1.6	14.8 ± 1.6	14.9 ± 1.5
Education, n, %				
<High school degree	1 (0.3)	1 (1.0)	0 (0.0)	0 (0.0)
High school degree	28 (9.2)	7 (6.9)	10 (9.8)	11 (10.8)
Some college	91 (29.8)	37 (36.6)	31 (30.4)	23 (22.6)
College degree	185 (60.7)	56 (55.5)	61 (59.8)	68 (66.7)
Current smoking, n (%)	31 (10.2)	9 (8.9)	13 (12.8)	9 (8.9)
Body mass index, m/kg^2^, median [quartile1, quartile3]	26.1 [23.2, 29.0]	26.7 [23.6, 30.3]	25.8 [23.2, 28.9]	25.6 [22.6, 28.6]
Systolic blood pressure mm Hg	117 ± 15	116 ± 15	119 ± 14	116 ± 15
Stage I hypertension, n (%)	57 (18.7)	12 (11.9)	27 (26.5)	18 (17.7)
Diabetes, n (%)	6 (2.0)	4 (4.0)	2 (2.0)	0 (0.0)
CES‐D score (16+), n (%)	36 (11.8)	15 (14.9)	8 (7.8)	13 (12.9)
Anti‐HTN medication, n (%)	29 (9.5)	6 (5.9)	15 (14.7)	8 (7.8)
HRT, n (%)	5 (2.6)	0 (0.0)	2 (2.7)	3 (4.8)
OCP, n (%)	61 (20.0)	14 (13.9)	9 (8.8)	38 (37.3)
APOE e4 positivity, n (%)	70 (23.8)	23 (23.5)	25 (25.3)	22 (22.7)
Camera, n (%); HR+ discovery GE‐smoothed	244 (80) 61 (20)	79 (78) 22 (22)	84 (82) 18 (18)	81 (79) 21 (21)
**Amyloid**				
Superior temporal sulcus	1.03 ± 0.06	1.03 ± 0.06	1.03 ± 0.06	1.03 ± 0.07
Precuneus amyloid, median [quartile1, quartile3]	1.12 [1.09, 1.17]	1.12 [1.09, 1.17]	1.13 [1.08, 1.17]	1.12 [1.09, 1.17]
Posterior cingulate amyloid	1.18 ± 0.12	1.19 ± 0.11	1.18 ± 0.11	1.18 ± 0.13
FLR, median [quartile1, quartile3]	1.05 [1.03, 1.08]	1.06 [1.03, 1.08]	1.05 [1.02, 1.09]	1.05 [1.02, 1.08]

**Tau**	(*N* = 261)	(*n* = 83)	(*n* = 84)	(*n* = 94)
Posterior cingulate tau	1.07 ± 0.07	1.08 ± 0.09	1.07 ± 0.07	1.06 ± 0.07
Entorhinal tau	1.05 ± 0.08	1.05 ± 0.08	1.04 ± 0.08	1.05 ± 0.07
Inferior temporal lobe tau	1.13 ± 0.07	1.15 ± 0.08	1.13 ± 0.07	1.11 ± 0.07
Fusiform gyrus tau	1.13 ± 0.07	1.14 ± 0.07	1.13 ± 0.07	1.11 ± 0.07
Precuneus tau	1.07 ± 0.07	1.08 ± 0.08	1.07 ± 0.07	1.06 ± 0.07
Parahippocampal	1.05 ± 0.07	1.06 ± 0.08	1.06 ± 0.07	1.05 ± 0.07
Amygdala	1.14 ± 0.11	1.15 ± 0.11	1.15 ± 0.11	1.13 ± 0.11
Insula	1.10 ± 0.08	1.11 ± 0.09	1.11 ± 0.08	1.09 ± 0.08
Temporal pole	1.07 ± 0.08	1.07 ± 0.09	1.06 ± 0.08	1.07 ± 0.07
Superior frontal orbital	1.01 ± 0.07	1.02 ± 0.08	1.01 ± 0.07	1.01 ± 0.07

*Note*: Values are mean ± standard deviation unless otherwise stated.

Abbreviations: APOE, apolipoprotein E; CES‐D, Center for Epidemiologic Studies Depression Scale; FLR, frontal‐lateral‐retrosplenial cortices; HRT, hormone replacement therapy; HTN, hypertension; PET, positron emission tomography; PiB, [^11^C]Pittsburgh Compound B;

### 2.7 Model specification for the analysis of the relationship between A‐beta and serum cortisol

We employed two covariate‐adjusted models for the analysis:

- Model 1 (M1): Adjusted for sex, age at the time of PET imaging, age‐squared (to account for non‐linearity), the interval between cortisol assessment and PET imaging, and scanner type.
- Model 2 (M2): Further adjusted for APOE4 positivity, defined as carrying one or more APOE4 alleles.

In line with prior research suggesting non‐linear effects of biological markers on disease risk, we categorized serum cortisol levels into tertiles. This approach accommodates potential U‐shaped associations, allowing us to examine whether extreme cortisol levels confer distinct risks for amyloid pathology. The middle tertile serves as a reference group, representing an optimal range for cortisol's physiological functions.

The primary outcomes for Aβ deposition were the DVR in the posterior cingulate, precuneus, and FLR cortices, with FLR values log‐transformed to address skewed distributions. Amyloid deposition in the STS was analyzed as a secondary outcome.

Model specification for the analysis of the relationship between tau and serum cortisol

For the tau imaging analysis, the primary outcomes included tau SUVRs in the inferior temporal gyrus, posterior cingulate, and entorhinal cortex. Three models were applied:

- Model 1 (M1): Adjusted for sex, age at the time of PET imaging, age‐squared (to account for non‐linearity), the interval between cortisol assessment and PET imaging, and scanner type.
- Model 2 (M2): Included additional adjustment for APOE4 status.
- Model 3 (M3): Further adjusted for FLR amyloid burden.

These models were also used for secondary outcomes, including tau SUVRs in the fusiform, precuneus, hippocampus, parahippocampal cortex, amygdala, insula, temporal pole, superior frontal orbital gyrus, and rhinal cortex. Interaction effects between serum cortisol and sex, menopause status (in women), and ApoE4 positivity were examined to explore potential modifying influences.

## 3 RESULTS

### 3.1 Participant characteristics

The sample consisted of 305 participants (48.5% female), with a mean age at the time of PiB PET scan of 54.7 ± 8.1 years. 23.8% were APOE4 positive. About 20% were receiving oral contraceptive therapy, and 2.6% were on hormone replacement therapy. 11.8% of participants met the criteria for depressive symptoms, with a score greater than 16 on the Center for Epidemiologic Studies Depression Scale (CES‐D). The median cortisol level was 13.2 µg/dL, within the physiological range. Only one participant was receiving corticosteroid therapy at the time of exam 1. The average times between cortisol sampling and PET scans for amyloid and tau were 15.0 and 14.8 years, respectively. The DVR and SUVR values for amyloid and tau are listed in Table 1.

### 3.2 Association between cortisol level and regional Aβ and tau

There was no association between serum cortisol levels (expressed categorically as tertiles) and regional amyloid DVR (Table 2). There was a statistically significant interaction between cortisol levels and sex in regional amyloid deposition in posterior cingulate (*p* = 0.018), precuneus (*p* = 0.009), and FLR (0.024) cortices. There was no interaction between cortisol level and STS amyloid (*p* = 0.281). The highest tertile of serum cortisol in women, compared to the middle tertile, was associated with higher amyloid deposition in the posterior cingulate cortex (PCC) (*β* ± SE: 0.065 ± 0.026, *p* = 0.013), precuneus (PC) (0.072 ± 0.025, *p* = 0.004), and FLR ROIs (0.039 ± 0.014, *p* = 0.006). The same relationship was not present in men (Tables 3 and 4 for Models 1 and 2, respectively). There was no interaction between APOE4 positivity and cortisol level with amyloid PET burden (all *p* > 0.729).

**TABLE 2:** Associations of serum cortisol and amyloid PET burden.

Parameter	Posterior cingulate	Precuneus	FLR^b^	Superior temporal sulcus
**Amyloid Burden & *Serum Cortisol β* ± SE (p)**				
*Continuous* ^a^				
Model 1	0.003 ± 0.008 (0.708)	0.008 ± 0.007 (0.295)	0.002 ± 0.004 (0.605)	−0.0003 ± 0.004 (0.950)
Model 2	0.001 ± 0.008 (0.920)	0.007 ± 0.008 (0.367)	0.001 ± 0.004 (0.806)	−0.0009 ± 0.004 (0.849)
*Tertiles*				
M1				
Tertile1	0.008 ± 0.016 (0.604)	0.004 ± 0.015 (0.766)	0.007 ± 0.009 (0.392)	0.007 ± 0.009 (0.454)
Tertile 2	Ref.	Ref.	Ref.	Ref.
Tertile 3	0.015 ± 0.016 (0.354)	0.021 ± 0.015 (0.164)	0.012 ± 0.009 (0.160)	0.008 ± 0.009 (0.396)
M2				
Tertile 1	0.012 ± 0.016 (0.439)	0.008 ± 0.015 (0.580)	0.010 ± 0.009 (0.233)	0.004 ± 0.016 (0.829)
Tertile 2	Ref.	Ref.	Ref.	Ref.
Tertile 3	0.013 ± 0.016 (0.404)	0.022 ± 0.015 (0.150)	0.012 ± 0.009 (0.166)	0.010 ± 0.017 (0.534)

*Notes*: M1: adjusted for age, age‐squared, time between cortisol assessment and PET, sex and camera. M2: adjusted for age, age‐squared, time between cortisol assessment and PET, sex, camera, and APOE4 positivity status.

Abbreviations: FLR, frontal‐lateral‐retrosplenial cortices; PET, positron emission tomography.

^a^ Square root transformed to normalize the skewed distribution.

^b^ Natural log transformed to normalize the skewed distribution.

**TABLE 3:** Associations of serum cortisol and amyloid PET burden: Interactions by sex.

Parameter	Model 1	Posterior cingulate	Precuneus^b^	FLR amyloid^b^	Superior temporal sulcus
**Amyloid Burden & *Serum Cortisol* Interaction by sex, in continuous & tertiles *β* ± SE (p)**					
*Continuous* ^a^					
	Interaction p	**0.024**	**0.050**	**0.043**	0.379
	Female	0.017 ± 0.010 (0.094)	0.019 ± 0.010 (0.054)	0.010 ± 0.006 (0.065)	–
	Male	−0.022 ± 0.014 (0.102)	−0.013 ± 0.012 (0.291)	−0.009 ± 0.008 (0.230)	–
*Tertiles*					
	Interaction p	**0.018**	**0.009**	**0.024**	0.281
	Female				
	T1	0.018 ± 0.025 (0.472)	0.018 ± 0.024 (0.458)	0.010 ± 0.013 (0.449)	–
	T2	Ref.	Ref.	Ref.	Ref.
	T3	**0.065 ± 0.026 (0.013)**	**0.072 ± 0.025 (0.004)**	**0.039 ± 0.014 (0.006)**	–
	*Male*				
	T1	0.006 ± 0.020 (0.763)	−0.002 ± 0.018 (0.909)	0.007 ± 0.011 (0.510)	–
	T2	Ref.	Ref.	Ref.	Ref.
	T3	−0.020 ± 0.020 (0.307)	−0.017 ± 0.018 (0.337)	−0.005 ± 0.011 (0.669)	–

*Note*: M1: adjusted for age, age‐squared, time between cortisol assessment and PET, sex and camera.

The statistically significant *p*‐values are in bold.

Abbreviations: FLR, frontal‐lateral‐retrosplenial cortices; PET, positron emission tomography.

^a^ Square root transformed to normalize the skewed distribution.

^b^ Natural log transformed to normalize the skewed distribution.

**TABLE 4:** Associations of serum cortisol and amyloid PET burden: Interactions by sex.

Parameter	Model 2	Posterior cingulate	Precuneus^b^	FLR amyloid^b^	Superior temporal sulcus
**Amyloid Burden & *Serum Cortisol* Interaction by sex, in continuous & tertiles *β* ± SE (p)**					
*Continuous* ^a^					
	Interaction p	**0.015**	**0.052**	**0.034**	0.417
	Female	0.015 ± 0.011 (0.146)	±0.010 (0.078)	0.009 ± 0.006 (0.113)	–
	Male	−**0.028 ± 0.013 (0.039)**	−0.015 ± 0.013 (0.239)	−0.012 ± 0.008 (0.129)	–
*Tertiles*					
	Interaction p	**0.018**	**0.015**	**0.027**	0.364
	Female				
	T1	0.021 ± 0.025 (0.416)	0.020 ± 0.024 (0.404)	0.013 ± 0.013 (0.338)	–
	T2	Ref.	Ref.	Ref.	Ref
	T3	**0.061 ± 0.026 (0.021)**	**0.071 ± 0.026 (0.007)**	**0.037 ± 0.014 (0.009)**	–
	Male				
	T1	0.010 ± 0.019 (0.591)	0.003 ± 0.018 (0.8890)	0.010 ± 0.011 (0.366)	–
	T2	Ref.	Ref.	Ref.	Ref
	T3	−0.024 ± 0.020 (0.220)	−0.016 ± 0.019 (0.383)	−0.006 ± 0.011 (0.584)	–

*Note*: M2: additionally adjusted for APOE4 positivity status.

The statistically significant *p* values are in bold.

Abbreviations: FLR, frontal‐lateral‐retrosplenial cortices; PET, positron emission tomography.

^a^ Square root transformed.

^b^ Natural log transformed to normalize the skewed distribution.

**FIGURE 1 alz70179-fig-0001:**
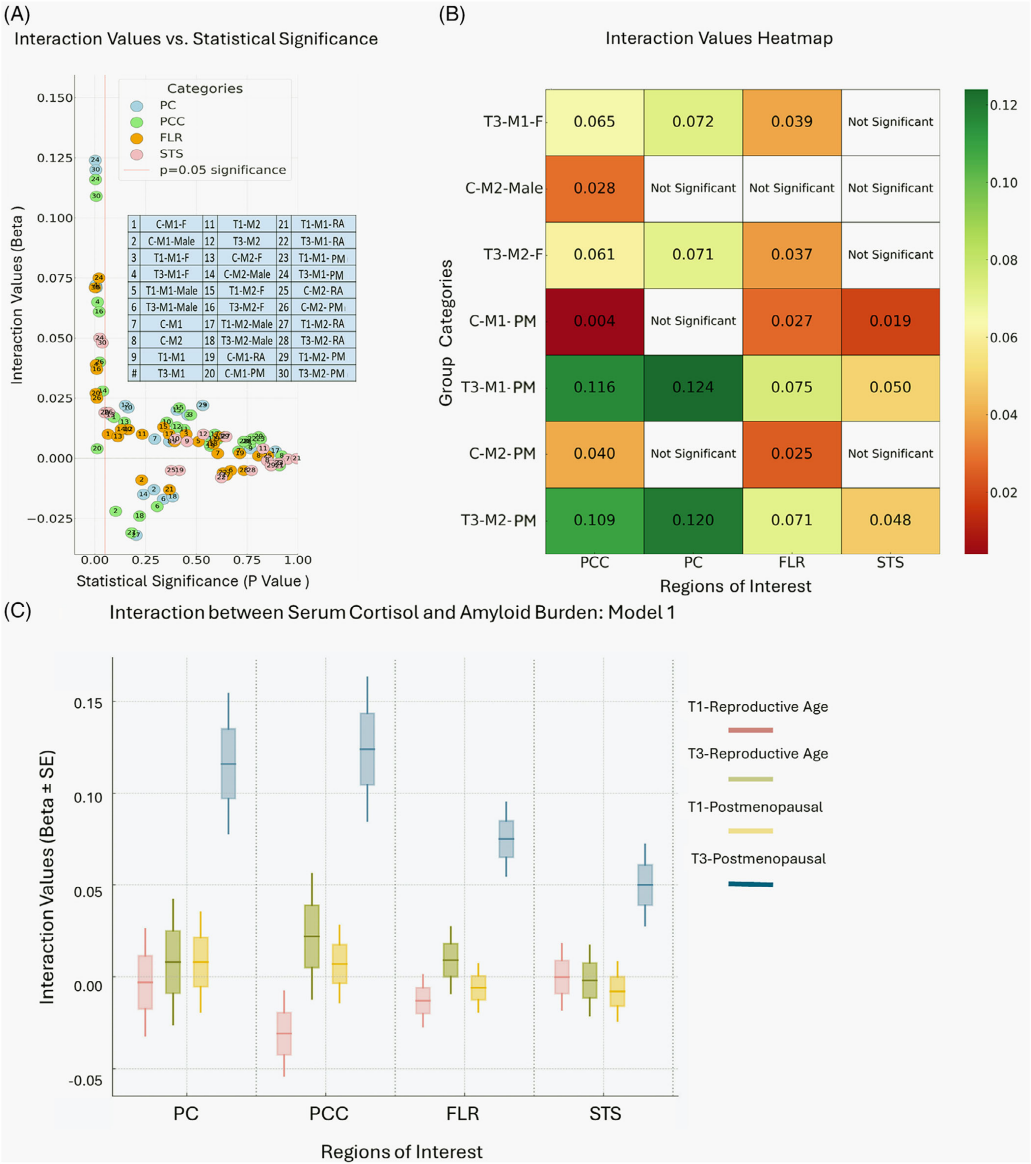
(A) The interactions values vs. statistical significance: This figure illustrates the interaction values and statistical significance of various combinations of cortisol levels, amyloid‐PET regions (PC, precuneus; PCC, posterior cingulate cortex; FLR, frontal‐lateral‐retrosplenial cortices, and STS, superior temporal sulcus), models (M1, M2), and participant subgroups (e.g., gender, menopausal status). Each data point (circle) is color‐coded by amyloid‐PET region, with significant interactions appearing to the left of the vertical red line at *p* = 0.05. The stratification by cortisol tertiles (T1: lowest, T3: highest), continuous data (C), and participant characteristics (e.g., F, female; RA, reproductive age; and PM, post‐menopause) provides insights into subgroup‐specific effects. The figure highlights statistically significant interactions while enabling comparisons across regions and groups. (B) Interaction values heatmap: This heatmap visualizes the numerical relationships between different categories (group categories listed on the y‐axis) and specific features (labeled on the x‐axis: PCC, PC, FLR, STS). It uses a color gradient from red to yellow to green, C‐interaction between serum cortisol and amyloid burden: interaction between serum cortisol levels and amyloid burden across regions of interest (PC, precuneus; PCC, posterior cingulate cortex; FLR, frontal lobe region; and STS, superior temporal sulcus) in Model 1. The interaction values (Beta ± SE) are stratified by menopausal status (reproductive age vs. post‐menopausal – PM) and cortisol tertiles (T1: lowest; T3: highest). Boxplots show the distribution of interaction values for each subgroup, with distinct colors corresponding to the specific combinations of cortisol levels and menopausal status as indicated in the legend. Positive interaction values suggest stronger associations, with notable differences observed across regions and subgroups.

There was an interaction by menopause status in the association between serum cortisol and amyloid PET burden in the PCC (*p* = 0.053), PC (*p* = 0.062) and FLR (*p* = 0.007). There was also an interaction by menopause status in the effect on the secondary ROI STS (*p* = 0.056). In post‐menopausal women only, we observed higher amyloid deposition in PCC (*β* ± SE: 0.116 ± 0.038, *p* = 0.003), PC (0.124 ± 0.039, *p* = 0.002), and FLR ROIs (0.075 ± 0.020, p ≤ 0.001) in those in the highest tertile cortisol compared to those in the middle tertile. Similarly, there was a greater amyloid burden in STS (0.050 ± 0.022, *p* = 0.024) in Tertile 3 for post‐menopausal women only. We did not find any significant relationships comparing the bottom tertile to the middle tertile (Tables 5 and 6). The Aβ related data are summarized in Figure 1.

**TABLE 5:** Associations of serum cortisol and amyloid PET burden in women (*n* = 148): Interactions by menopause status – Model 1.

Parameter	Model 1	Posterior cingulate	Precuneus^b^	FLR^b^	Superior temporal sulcus
**Amyloid Burden & *Serum Cortisol* Interaction by Menopause status *β* ± SE (p)**					
*Continuous* ^a^					
	Interaction p	**0.055**	0.279	**0.021**	**0.041**
	Pre‐menopause	0.005 ± 0.009 (0.570)	–	0.002 ± 0.005 (0.714)	−0.005 ± 0.006 (0.416)
	Menopause	**0.004 ± 0.017 (0.011)**	–	**0.027 ± 0.009 (0.005)**	**0.019 ± 0.001 (0.049)**
*Tertiles*					
	Interaction p	**0.053**	**0.062**	**0.007**	**0.056**
	Pre‐menopause				
	T1 (*n* = 19)	−0.003 ± 0.029 (0.909)	−0.031 ± 0.023 (0.181)	−0.013 ± 0.014 (0.367)	−0.0001 ± 0.018 (0.995)
	T2 (*n* = 12)	Ref.	Ref.	Ref.	Ref.
	T3 (*n* = 31)	0.008 ± 0.027 (0.774)	0.007 ± 0.021 (0.733)	−0.006 ± 0.013 (0.631)	−0.008 ± 0.016 (0.623)
	Menopause				
	T1 (*n* = 37)	0.008 ± 0.034 (0.816)	0.022 ± 0.034 (0.531)	0.009 ± 0.018 (0.609)	−0.002 ± 0.019 (0.907)
	T2 (*n* = 26)	Ref.	Ref.	Ref.	Ref.
	T3 (*n* = 23)	**0.116 ± 0.038 (0.003)**	**0.124 ± 0.039 (0.002)**	**0.075 ± 0.020 (< 0.001)**	**0.050 ± 0.022 (0.024)**

*Note*: M1: adjusted for age, age‐squared, time between cortisol assessment and PET, sex and camera.

The statistically significant *p* values are in bold.

Abbreviations: FLR, frontal‐lateral‐retrosplenial cortices; PET, positron emission tomography.

^a^ Square root transformed.

^b^ Natural log transformed to normalize the skewed distribution.

**TABLE 6:** Associations of serum cortisol and amyloid PET burden in women (*n* = 148): Interactions by menopause status – Model 2.

Parameter	Model 2	Posterior cingulate	Precuneus^b^	FLR^b^	Superior temporal sulcus
**Amyloid Burden & *Serum Cortisol* Interaction by Menopause status *β* ± SE (p)**					
*Continuous* ^a^					
	Interaction p	0.0996	0.350	**0.033**	**0.051**
	Pre‐menopause	0.006 ± 0.010 (0.566)	–	0.001 ± 0.005 (0.852)	−0.005 ± 0.006 (0.376)
	Menopause	**0.040 ± 0.018 (0.024)**	–	**0.025 ± 0.009 (0.010)**	0.019 ± 0.010 (0.068)
*Tertiles*					
	Interaction p	**0.0995**	**0.096**	**0.015**	**0.068**
	Pre‐menopause				
	T1 (*n* = 19)	−0.031 ± 0.023 (0.181)	−0.032 ± 0.025 (0.203)	−0.007 ± 0.016 (0.647)	0.009 ± 0.019 (0.646)
	T2 (*n* = 12)	Ref.	Ref.	Ref.	Ref.
	T3 (*n* = 31)	0.007 ± 0.021 (0.733)	0.007 ± 0.022 (0.749)	−0.005 ± 0.014 (0.734)	−0.005 ± 0.017 (0.771)
	Menopause				
	T1 (*n* = 37)	0.009 ± 0.035 (0.807)	0.022 ± 0.036 (0.532)	0.009 ± 0.019 (0.640)	−0.003 ± 0.020 (0.868)
	T2 (*n* = 26)	Ref.	Ref.	Ref.	Ref.
	T3 (*n* = 23)	**0.109 ± 0.039 (0.007)**	**0.120 ± 0.040 (0.004)**	**0.071 ± 0.021 (0.001)**	**0.048 ± 0.022 (0.036)**

Abbreviations: FLR, frontal‐lateral‐retrosplenial cortices; PET, positron emission tomography.

The statistically significant *p* values are in bold.

^a^ Square root transformed.

^b^ Natural log transformed to normalize the skewed distribution.

There was no association between cortisol and PET tau in the entorhinal, inferior temporal, and posterior cingulate cortices (primary analysis – see Table S1). Similarly, there was no association between serum cortisol levels and tau deposition in the precuneus, hippocampus, parahippocampal cortex, and amygdala (secondary outcomes). We did not observe any interaction by sex, APOE4 positivity status, or menopause status.

## 4 DISCUSSION

We used a retrospective review of longitudinal data from the FHS to examine the association between serum cortisol levels measured in middle age and amyloid/tau PET imaging outcomes approximately 15 years later in a well‐characterized cohort.

### 4.1 Summary of key findings

We observed a significant association between elevated serum cortisol in early midlife and increased amyloid deposition 15 years later, measured by PET imaging. This association was specific to post‐menopausal women and was absent in men or in relation to tau deposition in either sex. Although the effect size was modest, the association persisted even after controlling for age and other confounding variables. This finding aligns with the well‐established increased risk[Fig alz70179-fig-0001] and prevalence of AD in women, particularly following menopause. The lack of association with tau deposition also fits within the established timeline of AD pathology, where amyloid aggregation typically precedes tau pathology by years. The analysis revealed no significant difference between the middle and bottom tertiles, indicating that the impacts of cortisol at low and high extremes may differentially influence amyloid deposition. We also found no interaction with APOE4 status. We posit that cortisol affects Aβ metabolism and clearance by pathways that may be independent and non‐overlapping with APOE4‐related pathways. Importantly, our cohort was cognitively unimpaired, reflecting a preclinical AD stage where biomarkers are positive without symptoms. This focus aligns with the paradigm that AD progresses in two phases: a presymptomatic phase, where mechanisms like immune activity might be protective, and a symptomatic phase, where such mechanisms could accelerate progression.^31^, ^32^, ^33^, ^34^ Therefore, there is a strong argument for studying these phases separately. By excluding symptomatic patients, our study emphasizes the risk of developing AD rather than the rate of progression, distinguishing these as separate research questions.

### 4.2 Comparison with previous studies

Our study extends the existing literature by using a younger cohort (mean age: 39.6 years) compared to previous research, which often focused on older individuals who may already exhibit early signs of AD pathology. This younger demographic allows us to examine the role of cortisol as an early risk factor rather than a marker of disease progression. Additionally, employing sensitive biomarkers such as amyloid and tau PET imaging, instead of relying solely on clinical criteria or measures of cognitive decline, enhances our ability to detect very early disease‐related changes. Previous studies have shown that elevated cortisol levels correlate with faster cognitive decline, increased amyloid burden, and reduced brain volumes, particularly in older women. However, these findings have been largely cross‐sectional and in populations likely to include both preclinical and clinical AD cases, making it difficult to delineate cortisol's role as a risk factor from its involvement in disease progression. Our study addresses this gap by focusing on a well‐characterized, dementia‐free cohort, which allows us to better explore the early impacts of cortisol dysregulation on AD risk.

Our study found a difference in amyloid accumulation chiefly in the posterior cingulate and precuneus. The precuneus and posterior cingulate cortex emerge as primary sites of early amyloid deposition across multiple independent cohorts, including Alzheimer's Disease Neuroimaging Initiative (ADNI) and BioFINDER. This spatial pattern is consistently demonstrated through both cross‐sectional and longitudinal analyses, providing robust convergent evidence for the stereotyped progression of amyloid pathology in the human brain.^35^

### 4.3 Cortisol and cognitive decline

Earlier studies, beginning with Umegaki and colleagues,^36^ have demonstrated a link between elevated cortisol levels and faster AD progression, greater cognitive dysfunction,^5^, ^6^, ^7^, ^14^, ^15^, ^16^, ^17^, ^18^ and increased amyloid burden.^17^ Large prospective studies, such as the Baltimore Longitudinal Study of Aging, the Vienna Transdanube Aging Study, and the Longitudinal Aging Study Amsterdam, have also reported associations between elevated blood cortisol and future cognitive decline,^6^, ^7^ though some inconsistencies remain.^14^ Smaller longitudinal studies have similarly shown that higher cortisol levels correspond to faster cognitive decline.^15^, ^17^, ^18^ However, these cohorts often included older participants, many of whom may have had pre‐clinical AD at baseline, complicating the differentiation between cortisol as an AD risk factor and as a marker of disease progression.

### 4.4 Cortisol and amyloid pathology

One recent study cross‐sectionally examined the relationship between serum cortisol levels and amyloid PET scans in 277 cognitively normal middle‐aged individuals at risk for AD and found that higher cortisol levels were associated with higher Aβ load in AD‐vulnerable brain regions. Importantly, women exhibited stronger associations between cortisol and Aβ load. The results were consistent after adjusting for age, APOE epsilon 4 status, various health factors, and hormone therapy use.^21^ On the other hand, Wang et al. reported no significant link between cortisol and Aβ in CSF but identified associations with tau pathology and markers of synaptic degeneration, suggesting a potential role for cortisol in tau‐related neurodegeneration.^16^

Animal research supports the link between cortisol and amyloid accumulation, showing that when glucocorticoids are administered to non‐human primates, amyloid production increases,^19^ and when glucocorticoid receptors are blocked in mice using mifepristone, there are observable cognitive and biomarker improvements.^20^

### 4.5 Potential mechanisms linking cortisol to amyloid aggregation

Elevated cortisol might increase the amyloidogenic processing of APP by altering energy metabolism pathways, reducing synaptic resilience, or influencing inflammatory responses.^17^, ^19^, ^37^, ^38^ Stress triggers the release of corticotropin‐releasing factor (CRF), which raises Aβ levels through heightened neuronal activity.^39^, ^40^, ^41^ Cortisol also disrupts neuronal calcium homeostasis, making neurons more susceptible to Aβ toxicity and excitotoxic damage.^42^

### 4.6 Sex differences and mechanisms

We hypothesize that the sex‐specific associations observed in our study are due to inherent differences in corticosteroid physiology and the pathogenesis of AD between men and women. Variations in cortisol levels, stress responses, and hormone interactions likely contribute to the distinct risk profiles and disease progression patterns observed across sexes, especially in post‐menopausal women.^22^, ^23^, ^24^, ^43^, ^44^ Menopause is known to increase AD risk, likely due to the sharp decline in estrogen and progesterone. Reduced estrogen levels disrupt Wnt signaling, impair glucose metabolism, reduce synaptic protection, and decrease alpha‐secretase activity, promoting the amyloidogenic breakdown of amyloid precursor protein (APP).^45^, ^46^ Furthermore, sex differences in cortisol levels, diurnal rhythms, and responses to stress and adrenocorticosteroids are well‐documented.^44^, ^47^, ^48^, ^49^, ^50^ Interactions between sex hormones and corticosteroids throughout adulthood and during the development of the hypothalamus‐pituitary‐adrenal axis emphasize the complex role of cortisol in AD pathogenesis.^21^, ^51^, ^52^, ^53^ These sexually dimorphic pathways may make women more susceptible to cortisol dysregulation in the context of AD. Nonetheless, while our findings support a role for cortisol in AD pathogenesis, its causal involvement remains unproven and warrants further investigation. Understanding these sex differences is crucial for developing personalized prevention and treatment strategies for AD.^47^, ^48^, ^49^, ^50^, ^54^, ^55^, ^56^

### 4.7 Strengths and limitations

Our study has several strengths, including the use of amyloid and tau PET biomarkers and a well‐phenotyped, younger cohort, which allows for the investigation of early AD risk. However, our predominantly White sample limits the generalizability of our findings, and the reliance on a single morning cortisol measure may not fully capture long‐term dysregulation. Morning cortisol measurement was chosen by us because it captures the peak of the natural circadian rhythm when values are most stable, consistent, and less influenced by daily confounding variables.^57^, ^58^ This timepoint provides better technical reliability for laboratory assays, has established reference ranges for comparison, and is particularly sensitive to detecting HPA axis dysregulation associated with chronic stress. It is also cost‐effective and practical since it requires only a single time point collection rather than the multiple samples, specialized equipment, or laboratory processing needed for alternative methods like salivary diurnal profiles or hair cortisol analysis.^59^ This single measure limits our ability to differentiate chronic versus transient cortisol elevation, potentially influencing the observed associations. A better approach in future studies may be multiple 24‐h urinary cortisol measurements performed longitudinally.

The modest effect sizes (e.g., *β* ± SE: 0.116 ± 0.038 for PCC) suggest limited clinical impact at first. But to put this in perspective, using a *β* of 0.116, and p0 of 2.7%,^60^ a rough estimate of relative risk (RR) is 1.12 (RR = OR/(1 − p₀ + (p₀ × OR)), where the odds ratio (OR) = eβ. This is comparable with many established midlife risk factors for AD, such as physical inactivity (RR = 1.2), hypertension (1.2), or excessive alcohol consumption (1.2).^61^ Future research should incorporate more diverse populations and longitudinal cortisol measurements, such as 24‐h urinary or multiple salivary cortisol assessments, to better understand the chronic impact of cortisol. Additionally, studying the role of lifestyle and environmental factors, as well as potential mediators like inflammation or metabolic dysfunction, could shed further light on the pathways linking cortisol and AD risk. Future studies should also examine temporal lobe pathology as AD progresses.

### 4.8 Conclusion and future directions

Our findings suggest a potential sex‐specific mechanism linking cortisol dysregulation to early amyloid deposition, particularly in post‐menopausal women. The work reinforces the importance of considering sex and hormonal status in understanding AD pathogenesis and suggests that stress reduction and hormonal interventions may hold promise for AD prevention, especially in at‐risk women. Longitudinal follow‐up of our cohort will be crucial to determine whether these early amyloid changes translate into clinical symptoms and to clarify the causal role of cortisol in AD development. Given that our cohort exhibits Alzheimer's pathological change in a preclinical, presymptomatic stage – where biomarkers are positive without symptoms – longitudinal follow‐up is critical to determine if these amyloid changes predict clinical AD onset. This distinction between risk of onset and progression rate is vital for developing targeted interventions.

## AUTHOR CONTRIBUTIONS

Arash Salardini: Writing – original draft, review, editing, visualizations, and formal analysis. Jayandra J. Himali: Conceptualization, formal analysis – supervision, and review & editing. Muhammad S. Abdullah, Emer R. McGrath, Mitzi M. Gonzales, Emma G. Thibault, Joel Salinas, Hugo J. Aparicio, Rachel F. Buckley, Claudia L. Satizabal, and Vanessa Young: Writing – review & editing. Rima Chaudhari: Writing, review, and analysis. Eduardo M. Zilli and Ramachandran S. Vasan: Conceptualization. Dibya Himali: Formal analysis. Saptaparni Ghosh: Data curation. Keith A. Johnson, Charles DeCarli, and Georges El Fakhri: Resources and data curation. Alexa S. Beiser: Conceptualization and methodology. Sudha Seshadri: Conceptualization; funding acquisition and supervision.

## CONFLICT OF INTEREST STATEMENT

Arash Salardini, declares honoraria from Eli Lilly Inc. Jayandra J. Himali, has received institutional funding from the National Institutes of Health (NIH), the Alzheimer's Drug Discovery Foundation, and the Alzheimer's Association. Muhammad S. Abdullah, Rima Chaudhari, Vanessa Young, Eduardo M. Zilli, Emma G. Thibault, Dibya Himali, Saptaparni Ghosh, Claudia L. Satizabal, and Ramachandran S. Vasan declare no conflicts of interest. Emer R. McGrath has received institutional funding from the NIH and the American Academy of Neurology. Mitzi M. Gonzales has received institutional funding from the National Institute on Aging and support from Biogen. Joel Salinas has received institutional funding from the NIH‐NIA, honoraria from Eli Lilly Inc., and consulting fees from Eisai, Inc., and has a patent pending. Hugo J. Aparicio has received institutional funding from the NIH and the American Academy of Neurology. Rachel F. Buckley has received institutional funding from the NIA and the Alzheimer's Association and honoraria from UT Dallas. Keith A. Johnson has received institutional funding from the NIH and consulting fees from Merck, and serves unpaid on an advisory board for Cerveau. Charles DeCarli has received institutional funding from the NIH. Georges El Fakhri has received institutional funding from the NIH. Alexa S. Beiser has received institutional funding from the NIH, the Alzheimer's Drug Discovery Foundation, and the Alzheimer's Association. Sudha Seshadri has received institutional funding from the NIH, the Alzheimer's Association, and the Alzheimer Drug Discovery Foundation. Author disclosures are available in the Supporting Information.

## CONSENT STATEMENT

This study was conducted using de‐identified data from the Framingham Heart Study, a publicly available dataset. The use of these data was approved by Boston University and Mass General Hospitals Institutional Review Boards (IRBs), ensuring compliance with the Declaration of Helsinki and relevant regulations. Participants of the Framingham Heart Study provided informed consent at the time of data collection, permitting their data to be used for research purposes. No additional consent was required for this secondary analysis as the data were fully anonymized.

## Supporting information



Supporting Information

Supporting Information
