# Supplementary material for: Elevated serum cortisol associated with early‐detected increase of brain amyloid deposition in Alzheimer's disease imaging biomarkers among menopausal women: The Framingham Heart Study
Source: Alzheimers Dement. 2025 Apr 24;21(4):e70179. doi: 10.1002/alz.70179 (PMC12019305; doi:10.1002/alz.70179)
Supplement: Supplementary file 1 — Supporting Information [file ALZ-21-e70179-s002.docx]

**S1: Associations of serum cortisol and tau PET burden**

|  | Entorhinal tau | Inferior temporal lobe tau | Posterior cingulate tau | Precuneus | Hippocampus | Parahippocampal | Amygdala | Insula | Temporalpole | Superiorfrontal | Fusiform | Rhinal |
| --- | --- | --- | --- | --- | --- | --- | --- | --- | --- | --- | --- | --- |
|  | β±SE (p) | β±SE (p) | β±SE (p) | β±SE (p) | β±SE (p) | β±SE (p) | β±SE (p) | β±SE (p) | β±SE (p) | β±SE (p) | β±SE (p) | β±SE (p) |
| T1(Model 1) | 0.012±0.012 (0.354) | 0.019±0.011 (0.086) | 0.011±0.011 (0.343) | 0.014±0.011 (0.175) | 0.012±0.016 (0.452) | 0.008±0.011 (0.466) | 0.025±0.016 (0.117) | 0.019±0.012 (0.132) | 0.004±0.013 (0.764) | 0.008±0.011 (0.486) | 0.019±0.010 (0.073) | 0.014±0.014 (0.309) |
| T2(Model 1) | Ref | Ref | Ref | Ref | Ref | Ref | Ref | Ref | Ref | Ref | Ref | Ref |
| T3(Model 1) | 0.002±0.012 (0.880) | -0.008±0.011 (0.437) | -0.008±0.011 (0.454) | -0.004±0.010 (0.726) | -0.015±0.016 (0.349) | -0.006±0.011 (0.603) | -0.0003±0.016 (0.985) | -0.008±0.012 (0.499) | 0.0007±0.013 (0.958) | -0.011±0.011 (0.325) | 0.002±0.010 (0.878) | 0.002±0.014 (0.879) |
| T1(Model 2) | 0.011±0.013 (0.403) | 0.020±0.011 (0.082) | 0.011±0.012 (0.335) | 0.014±0.011 (0.201) | 0.011±0.017 (0.503) | 0.008±0.011 (0.495) | 0.024±0.016 (0.145) | 0.018±0.013 (0.147) | 0.004±0.013 (0.782) | 0.007±0.011 (0.537) | 0.019±0.011 (0.070) | 0.013±0.014 (0.358) |
| T2(Model 2) | Ref | Ref | Ref | Ref | Ref | Ref | Ref | Ref | Ref | Ref | Ref | Ref |
| T3(Model 2) | 0.003±0.012 (0.790) | -0.007±0.011 (0.548) | -0.005±0.011 (0.670) | -0.003±0.011 (0.798) | -0.014±0.017 (0.390) | -0.004±0.011 (0.733) | 0.0004±0.016 (0.978) | -0.007±0.012 (0.584) | 0.001±0.013 (0.945) | -0.008±0.011 (0.449) | 0.003±0.011 (0.760) | 0.001±0.014 (0.939) |
| T1(Model 3) | 0.009±0.012 (0.463) | 0.019±0.011 (0.100) | 0.010±0.011 (0.392) | 0.012±0.011 (0.242) | 0.011±0.017 (0.526) | 0.006±0.011 (0.576) | 0.023±0.016 (0.156) | 0.018±0.013 (0.161) | 0.003±0.013 (0.837) | 0.006±0.011 (0.610) | 0.019±0.012 (0.077) | 0.012±0.014 (0.396) |
| T2(Model 3) | Ref | Ref | Ref | Ref | Ref | Ref | Ref | Ref | Ref | Ref | Ref | Ref |
| T3(Model 3) | 0.002±0.012 (0.906) | -0.008±0.011 (0.458) | -0.007±0.011 (0.558) | -0.004±0.011 (0.679) | -0.015±0.017 (0.367) | -0.006±0.011 (0.603) | -0.0003±0.016 (0.986) | -0.008±0.012 (0.542) | -0.0004±0.013 (0.977) | 0.010±0.011 (0.362) | 0.003±0.011 (0.780) | -0.0004±0.014 (0.979) |

Model 1 (M1): adjusted for age, age-squared, time between cortisol assessment and PET, sex and camera

Model 2 (M2): adjusted for age, age-squared, time between cortisol assessment and PET, sex, camera and ApoE4.

Model 3 (M3): adjusted for age, age-squared, time between cortisol assessment and PET, sex, camera, ApoE4, and FLR amyloid.
